# CRISPR/Cas9 establishment-mediated targeted mutagenesis in *Macrobrachium nipponense*

**DOI:** 10.3389/fphys.2023.1141359

**Published:** 2023-03-24

**Authors:** Hui Qiao, Sufei Jiang, Hongtuo Fu, Yiwei Xiong, Wenyi Zhang, Lei Xu, Dan Cheng, Jisheng Wang

**Affiliations:** ^1^ Key Laboratory of Freshwater Fisheries and Germplasm Resources Utilization, Ministry of Agriculture and Rural Affairs, Freshwater Fisheries Research Center, Chinese Academy of Fishery Sciences, Wuxi, China; ^2^ Wuxi Fisheries College, Nanjing Agricultural University, Wuxi, China

**Keywords:** CRISPR/Cas9, *Macrobrachium nipponense*, *vitellogenin*, *eyeless*, microinjection

## Abstract

**Introduction:** CRISPR/Cas9 is a gene-editing technology which could specifically cleave dsDNA and induce target gene mutation. CRISPR/Cas9 has been widely used in gene functional studies in many fields, such as medicine, biology, and agriculture due to its simple design, low cost, and high efficiency. Although it has been well developed in model fish and freshwater fish for gene function analysis, it is still novel in the studies dealing with economic crustacean species.

**Methods:** In this study, we established a CRISPR/Cas9 system based on microinjection for *M. nipponense*, an important economic crustacean aquaculture species. The *vitellogenin* (*Vg*) gene and the *eyeless* (*Ey*) gene were selected as the targeted genes for mutation. Two sgRNAs were designed for *Mn-Vg* and *Mn-Ey* gene editing, respectively.

**Results and Discussion:** For sg-*Vg*-1, the gastrula survival ratio was 8.69%, and the final hatching ratio was 4.83%. The blastula mutant ratio was 10%, and the hatching individual mutant ratio was 30%. For sg-*Vg*-2, the gastrula survival ratio was 5.85%, and the final hatching ratio was 3.89%. The blastula mutant ratio was 16.67%, and no mutant sequences were detected in hatching individuals. For sg-*Ey*-1, the gastrula survival ratio was 6.25%, and the final hatching ratio was 2.34%. The blastula mutant ratio was 10.00%, and the hatching individual mutant ratio was 66.67%. For sg-*Ey*-2, the gastrula survival ratio was 6.00%, and the final hatching ratio was 2.67%. No mutant sequence was detected in both blastula stage and hatching individuals. There were no significant morphological changes observed in the *Mn-Vg* group. Two deformed types were detected in sg-*Ey*-1-injected embryos. An evident developmental delay of the compound eye was detected in *Ey-*sg1-H1 in the zoea stage. The compound eyes of the *Ey-*sg1-H2 embryo could not form well-defined spheres, and the whole compound eye appeared to diffuse at the end of the late zoea stage. The establishment of a gene-editing platform based on CRISPR/Cas9 will not only provide an efficient and convenient method for gene function analysis but also provide a powerful tool for molecular-assisted breeding of *Macrobrachium nipponense*.

## 1 Introduction

*Macrobrachium nipponense*, commonly known as the oriental river prawn, is deeply favored by consumers in China due to its tender flesh, flavor, and high nutrient value (Fu et al., 2004; Cai and Shokita, 2006). In China, the culturing of this prawn started in the mid-1960s. At the end of the 1990s, its market price in East China reached 90 yuan/kg, with the demand in short supply, until recently. With the improvement in living standards, consumer demand for prawns has been increasing. However, the large-scale fishing and unscientific farming modes of prawns have caused the rapid degradation of *Macrobrachium nipponense* populations. By the end of the 1990s, severe varietal degradation was prevalent, which was reflected in early sexual maturity, individual miniaturization, commodity rate decline, and increased diseases, among others (Fu et al., 2012; Fu and Jin, 2018). As a result, the benefit of prawn culture has been significantly decreased, and the culture risk has increased. Therefore, it is imperative to carry out genetic improvement in *M. nipponense*. Since 2002, the present authors have been mainly engaged in the cultivation of a new variety of *M. nipponense*. Over the past 20 years, three new varieties of *M. nipponense* (hybrid *M. nipponense* “Taihu No.1,” *M. nipponense* “Taihu No.2,” and *M. nipponense* “Taihu No.3”) have been obtained based on hybrid breeding and classical selective breeding methods. These new varieties have passed the examination and approval of the national aquatic products commission and have been demonstrated and promoted on a large scale, covering more than 10 provinces in China. The cultivation and promotion of new varieties effectively alleviate the bottleneck of variety degradation and produce significant economic and social benefits. According to the statistics of the China Fishery Yearbook, the annual production of *M. nipponense* in 2021 was almost 250,000 t (Ministry of Agriculture Fisheries Bureau, 2021). Prawn culturing has become one of the important approaches for improving agricultural efficiency and increasing farmers’ income. However, the cycles of hybrid breeding and classical selective breeding methods are long. In order to improve the production of commercial shrimp and the farming breeding efficiency, additional new varieties with different growth characteristics should be rapidly cultivated to meet the growing market demand.

Gene-editing technology based on the CRISPR/Cas9 system (the clustered regularly interspaced short palindromic repeats and their associated endonuclease 9) is the artificial modification of genes at specific target sites with the help of an endonuclease, which can completely inactivate target genes with almost no species restriction (Jinek et al., 2012). In recent research studies, CRISPR/Cas9 has been broadly applied to animal and crop improvement due to its remarkable advantages of simplicity and efficiency (Baek et al., 2016; Dong et al., 2016; Kim et al., 2018; Johansen et al., 2019; Alward et al., 2020). With the continuous development of gene-editing technology, CRISPR/Cas9 has recently been applied to studies of gene function in aquatic animals. In fish, efficient CRISPR/Cas9 gene-editing systems have been established early in zebrafish (*Danio rerio*) and medaka (*Oryzias latipes*) (Jao et al., 2013; Ansai and Kinoshita, 2014). In *Salmo salar*, tyrosinase (*tyr*) gene editing mediated by CRISPR/Cas9 revealed its role in pigment formation (Edvardsen et al., 2014). CRISPR/Cas9 has also been introduced to crustaceans with many attempts in *Daphnia magna*, *Exopalaemon carinicauda*, *Neocaridina heteropoda*, *Eriocheir sinensis*, and *Macrobrachium rosenbergii* (Nakanishi et al., 2014; Gui et al., 2016; Li et al., 2022; Molcho et al., 2022). Some preliminary breakthroughs have been made, which showed exciting prospects for crustacean aquaculture. However, CRISPR/Cas9 gene editing has not been applied to *M. nipponense* yet.

In this study, a gene-editing platform mediated by CRISPR/Cas9 in *M. nipponense* was established by microinjection. The *vitellogenin* (*Vg*) gene and *eyeless* (*Ey*) gene were used to build a targeted mutagenesis system. The establishment of a gene-editing platform based on CRISPR/Cas9 will not only provide an efficient and convenient method for gene function analysis but also deliver a powerful tool for the molecular-assisted breeding of *M. nipponense*.

## 2 Materials and methods

### 2.1 Prawn rearing and embryo collection

The experiment was conducted from May to June 2022 at a water temperature of 25°C ± 2°C. Healthy adult male and female *M. nipponense* prawns with mature gonads (*n* = 50; body weight BW ± SD: 1.81 ± 0.46 g) were selected from the Dapu scientific experimental base at the Freshwater Fisheries Research Center (Wuxi, China). All prawns were cultured in a recirculating aquarium system. Male and female prawns mated under natural conditions and laid eggs, and then, the female prawns held the sticky eggs on their abdomen until the larvae hatched. The sticky single-celled embryos were collected after prawning and were carefully and gently separated into dispersed units with sterilized tweezers. All the separated embryos were then placed in a 10-ml Petri dish with sterilized fresh water and kept at a room temperature of 27°C ± 1°C before microinjection.

### 2.2 *Vitellogenin* and *eyeless* gene screening and structure analysis

The cDNA sequence of *Mn-Vg* was obtained from GenBank using the accession number KJ768657. The partial cDNA sequence of *Mn-Ey* was obtained from eyestalk transcriptomes of *M. nipponense* (NCBI, PRJNA339889). Two cDNA sequences were aligned to the reference genome of *M. nipponense* (NCBI accession number.: ASM1510439v1, https://ftp.cngb.org/pub/CNSA/data2/CNP0001186/CNS0254395/CNA0014632/) to obtain total gene sequences, and exon and intron locations were determined by sequence alignment analysis. About 0.1 g of muscle from one prawn was used for DNA extraction as described in a previous study (Jiang et al., 2019). Partial sequences of *Mn-Vg* and *Mn-Ey*, which were used as targets, were validated using the primers listed in Table 1. All sequences validated in this study were sequenced by Sangon Biotech (Shanghai, China). DNAMAN 6.0 was used for sequence comparison, and MEGA 7.0 (MEGA) was used to perform a neighbor-joining phylogenetic tree (Wang et al., 2019).

**TABLE 1:** Primers used in this study.

Primer/gRNA	Primer sequence (5′–3′)
sg-*Vg*-1	TAG​CTT​CCA​TGC​CCT​TCG​TAC​T
sg-*Vg*-2	GTT​GAG​TTG​TAC​CCA​TTC​TCT​GA
sg-*Ey*-1	CCT​CCA​GCT​CCT​GCA​GTA​GCC​A
sg-*Ey*-2	GGC​GTG​TTC​GAA​TTA​CTG​CTG​C
sg-*GFP*	GAT​CAT​ATG​AAA​CGG​CAT​GAC​T
*eIF*-F (qPCR)	CAT​GGA​TGT​ACC​TGT​GGT​GAA​AC
*eIF*-R (qPCR)	CTG​TCA​GCA​GAA​GGT​CCT​CAT​TA
*Ey*-F (qPCR)	CAG​GAC​CCC​ATG​TAC​GAT​AAA​CT
*Ey*-R (qPCR)	CAA​GCT​GTC​AAT​CTG​ATC​GTT​GG
*Vg*-F (qPCR)	GAA​GTT​AGC​GGA​GAT​CTG​AGG​T
*Vg*-R (qPCR)	CCT​CGT​TGA​CCA​ATC​TTG​AGA​G
*Vg*-F	CTC​ACC​TAA​GAA​AGC​TAC​TGC​CA
*Vg*-R	CAC​TTG​CCA​TTC​AAC​TCC​CAT​TT
*Ey*-F	CCG​TAG​GTG​TCG​TCC​ATC​AAC
*Ey*-R	GAC​AGA​CAC​GAA​AGG​ATC​CGA

### 2.3 Expression profiles of *Mn-Vg* and *Mn-Ey* in embryo development stages

The expression profiles of both genes in different embryo development stages were detected using qPCR. The embryo development stages were divided according to the previous studies, including CS (cleavage stage), BS (blastula stage), GS (gastrulation stage), NS (nauplius stage), ZS (zoea stage), and L1 (the first day larvae after hatching) (Qiao et al., 2015). Samples from each stage were collected under the microscope based on morphological characteristics and then placed in liquid nitrogen for RNA extraction. RNA was extracted using RNAiso Plus reagents, and the M-MLV reverse transcriptase kit was used for first-strand cDNA synthesis (Takara, Japan). The amplification procedure of qPCR was followed as described in previous studies, with eIF gene as the internal reference gene, and data were calculated by using the 2^−ΔΔCT^ method and SPSS 23.0 (Hu et al., 2018). One-way ANOVA and two-tailed Student’s *t*-tests were applied, and differences were considered significant at *p* < 0.05.

### 2.4 sgRNA preparation and *in vitro* digestion

The gRNA were designed through online software (http://zifit.partners.org/ZiFiT/ChoiceMenu.aspx) (Table 1), and partial coding genomic sequences were used as the template. The *GFP* gene was employed as a control (GenBank accession number: AF324408.1). The sgRNAs of both *Mn-Vg–Mn-Ey* and *GFP* were synthesized using the TranscriptAid T7 High Yield Transcription Kit (Thermo Scientific, United States of America) and were purified by ethanol precipitation; then, they were dissolved in DEPC-treated water. The fragment size and quality of sgRNAs were determined by 1.5% agarose gel electrophoresis, and the concentration was determined using the Nanodrop ND-2000.

An *in vitro* digestion was processed to evaluate the editing efficiency. Partial DNA, including the target loci of *Mn-Vg* and *Mn-Ey* separately, was amplified using the special primers listed in Table 1. Amplification reactions were carried out in a final volume of 25 μl containing forward and reversed primers (10 μmol/l) 1 μl, PCR mix 12.5 μl, genomic DNA template (50 ng/μl) 2 μl, and ddH_2_O 8.5 μl. PCR conditions were follows: initial denaturation at 94°C for 5 min; 30 cycles of 94°C for 30 s, 55°C for 30 s, and 72°C for 45 s; and finally, 72°C for 10 min. A Cas9 protein (Thermo Fisher Scientific, United States of America; A50575) was used for digestion, and the concentrations of Cas9 protein and sgRNA were 250 ng/μL and 100 ng/μL, separately. Both Cas9 protein and sgRNA were mixed and incubated at 37°C for 5 min. Afterward, 50 ng DNA of PCR products was digested using 1 μl complex of sgRNA and Cas9. The digestion was performed in a thermal cycler with the following conditions: 37°C for 1 h, 80°C for 5 min, and finally, 4°C for 10 min. Then, the digested products were evaluated by 2% agarose gel electrophoresis.

### 2.5 *Macrobrachium nipponense* embryo microinjection

A TransferMan 4r and a FemtoJet 4i microinjector (Eppendorf, Germany) were employed to perform embryo microinjection in *M. nipponense* with commercial Femtotip II microcapillaries (Eppendorf, Germany). The separated embryos in the one-cell stage were placed and arranged in fluted agar plates, and microinjection was carried out under the S9D stereomicroscope (Leica, Germany) at a room temperature of 27°C ± 1°C. The injected fluid comprised the mixture of sgRNA and Cas9 protein, similar to the mixture used in the *in vitro* digestion. The injection volume was set at approximately 0.5 nL. The embryo control group was injected with the same volume of the mixture of sg-*GFP* and Cas9 protein. The injected and control embryos were transferred and incubated in 10-cm-diameter disposable Petri dishes with sterilized fresh water, and the incubated water was changed twice a day.

### 2.6 Mutation detection

The blastula and hatching larva stages were selected for sampling. These two stages are the representative period of *M. nipponense* embryonic development. The fertilized egg, cleavage, and blastula stages of the embryo all belonged to the cell division phase, from single cell to multicellular. Hence, the blastula stage with more cells was selected as the representative sample stage. The embryo enters the tissue differentiation and organogenesis stage since the gastrula stage, and fully differentiated larvae were chosen as another representative sample stage. In a mixed embryo mutation analysis, since prawn embryos are very small (5–600 μm) in diameter, the unhatched injected embryos were tested by mixing 30 of them in a group. In a single embryo mutation analysis, injected larvae after hatching were used as templates individually for detection. DNA extraction and PCR methods were performed as described in Section 2.4. The PCR product was purified using the DiaSpin DNA Gel Extraction Kit (Sangon, China), then ligated to the pMD18-T vector (Takara, Japan), and transformed into *E.coli* DH5α competent cells (Takara, Japan; 9057). Thirty positive clones were selected by blue–white spot screening for sequencing by Shanghai Sangon Company (Sangon, China).

## 3 Results

### 3.1 Embryo stage identification and optimal collection time for microinjection

**FIGURE 1:**
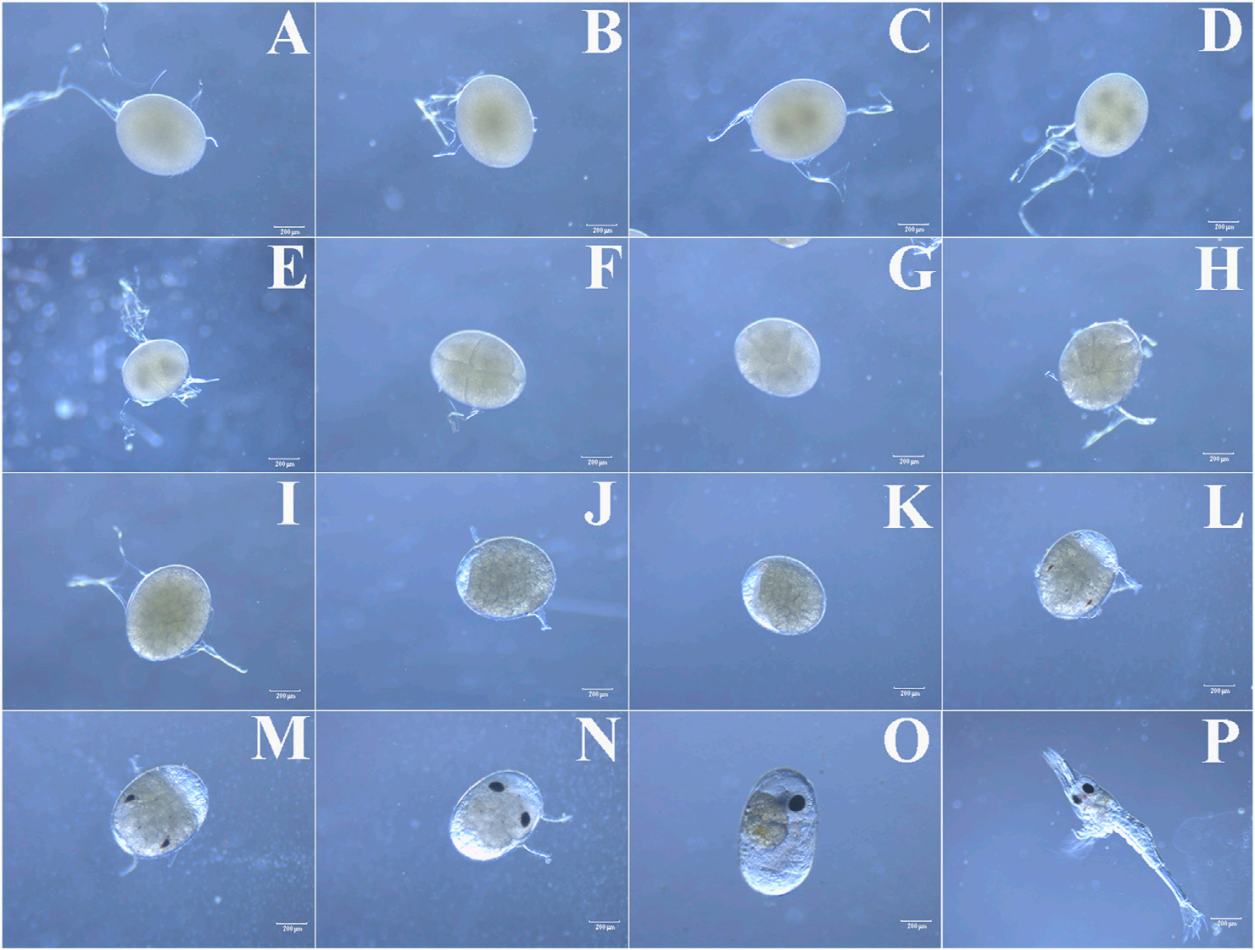
**(A)** Fertilized egg, **(B)** one-nucleus stage, **(C)** two-nucleus stage, **(D)** four-nucleus stage, **(E–H)** cleavage stage, **(I)** blastula stage, **(J)** gastrula stage, **(K)** nauplius stage, **(L)** early-zoea stage, **(M, N, O)** late- zoea stage and **(P)** hatching larval stage.

In order to accurately grasp the optimal time of microinjection, we determined the different stages of *M. nipponense* embryo development by microscopic observations at 27°C ± 1°C. According to the characteristics, embryos can be divided into fertilized egg, cleavage, blastula, gastrula, nauplius, zoea, and hatching larvae stages (Figure 1). The size of the newly produced fertilized egg (one-cell stage) was between 500 and 600 μm in diameter. The surface of the embryo was smooth with a secondary membrane (mucin), and it was filled with yolk. At this time, there was no evident nucleus, and this period lasted for about 1 h (Figure 1A). A nucleus was first observed in the center of the embryo approximately 1 h after ovulation. The one-nucleus stage lasted for about 1 h, and then, the nucleus divided into two (Figure 1B). The two-nucleus stage lasted for about 1.5 h, and the two nuclei divided into four (Figure 1C). The four-nucleus stage lasted no more than 0.5 h, and then, the embryos entered the cleavage stage (Figure 1D). A transverse cleavage formed initially with a clear cleavage furrow, followed by a longitudinal cleavage (Figures 1E,F). There was no evident division inside the embryo. Then, the nucleus gradually migrated from the center of the embryo to the surface, and the cell membrane depressed inward to form independent cells (Figures 1G,H). The embryos then entered a vigorous stage of division (blastula stage), and the number of cells increased rapidly. Tiny irregularly shaped cleavage cells were tightly packed on the surface of the embryos (Figure 1I). The blastula stage lasted for about 48 h, and then, the embryos entered the gastrula stage, where a clear zone appeared at one end of the embryos (Figure 1J). The gastrula stage lasted for approximately 1 day, and the clear zone gradually increased and the embryo began to enter the nauplius stage (Figure 1K). The nauplius stage lasted for about 3 days, with the clear zone gradually increasing to half the size of the entire embryo. The embryo then entered the zoea stage. The appearance of the compound eye pigment indicated this stage (Figure 1L), which lasted for about 9 days. As the embryos developed, the pigment bands became thicker, from half the size to the whole compound eye, changing from red to black in color (Figures 1M–O). The body segments were gradually formed, the yolk was depleted, and the internal structure gradually became clear. After about 15 days of embryo development, the larvae hatched (Figure 1P). The embryos used for microinjection were collected at the one-cell stage (fertilized egg stage). Since the four-nucleus stage lasted no more than 0.5 h and the embryos quickly entered the cleavage stage, collecting embryos before 3.5 h post-spawning was suggested to be the optimal time for microinjection based on the aforementioned results.

### 3.2 Genomic sequence information and expression profiles of *Mn-Vg* and *Mn-Ey*

**FIGURE 2:**
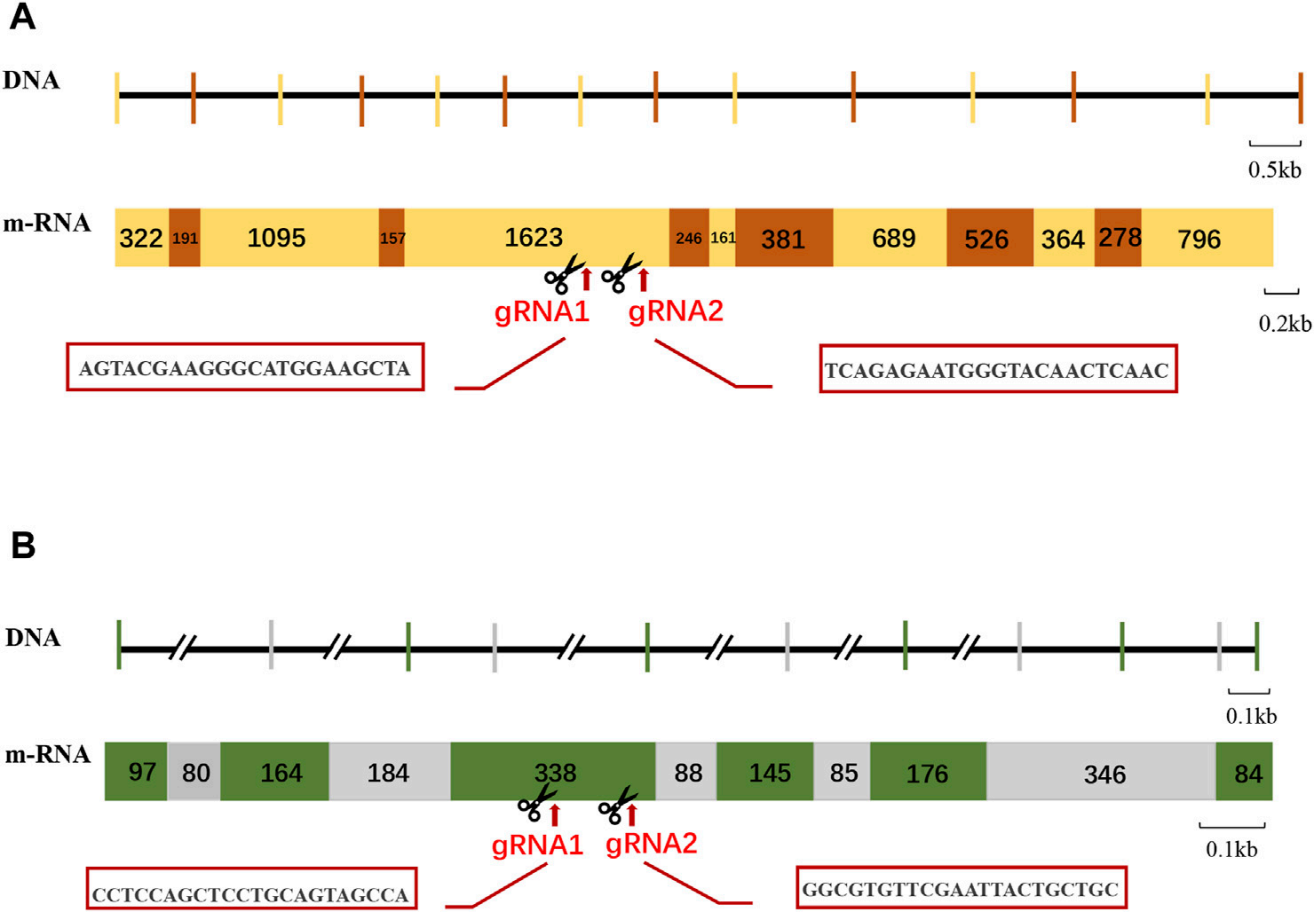
Schematic structure and gRNA site of *Mn-Vg* **(A)** and *Mn-Ey* **(B)** genes. For the DNA level: The Horizontal lines represent the intron sequences, and vertical lines represent the exon sequence. The long introns are represented by double slants. For the mRNA level: The rectangles with numbers represent the mRNA structures of genes. The number means the length of exons. The gRNA (guide RNA) site is indicated by arrows, and the sequences are shown in the red box.

The cDNA sequences of *Mn-Vg* and *Mn-Ey* were employed comparing with the genome of *M. nipponense* to identify the genomic sequence structure; two genes were submitted to NCBI (GenBank accession numbers: OP924286 and OP924287). The exons and introns were determined by the GU–AG rule. The complete genomic sequence of *Mn-Vg* was 10,038 bp, encoding 2,598 amino acids. In total, 12 introns and 13 exons were identified in the *Mn-Vg* genomic sequence. The full-length genomic sequence of *Mn-Ey* was 52,840 bp, encoding 350 amino acids. There were 10 introns and 11 exons detected in the *Mn-Ey* genomic sequence. Among these six introns, intron-1 and intron-2 were very long with the length of 9,814 bp and 38,266 bp, respectively. The sequence structures of *Mn-Vg* and *Mn-Ey* are shown in Figures 2A,B.

**FIGURE 3:**
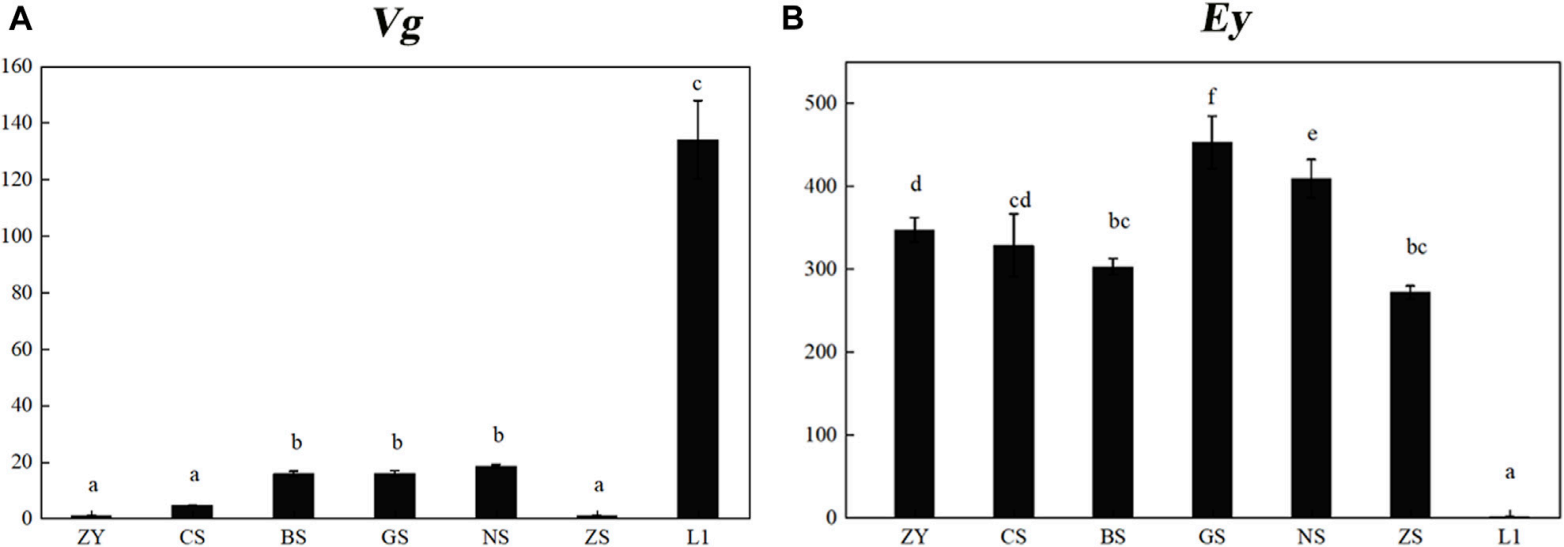
Expression profiles of *Mn-Vg* **(A)** and *Mn-Ey* **(B)** genes in embryo stages. ZY, zygote stage; CS, cleavage stage; BS, blastula stage; GS, gastrula stage; NS, nauplius stage; ZS, zoea stage; L1, the first day of the hatching larval stage. Data are shown as mean ± SD (*n* = 5). Different letters denote significant differences (*p* < 0.05).

The expression profiles of *Mn-Vg* and *Mn-Ey* during the zygote, cleavage, blastula, gastrula, nauplius, zoea, and hatching larvae stages were examined by qPCR. The results showed that *Mn-Vg* expression was very low in the early embryo stages, and it significantly increased after the larvae hatching stage (Figure 3A). The results of *Mn-Ey* expression were just the opposite of *Mn-Vg*. The expression of *Mn-Ey* was relatively high in the embryo stages, while it reduced significantly when the larvae hatched (Figure 3B).

### 3.3 sgRNA design and *in vitro* digestion

**FIGURE 4:**
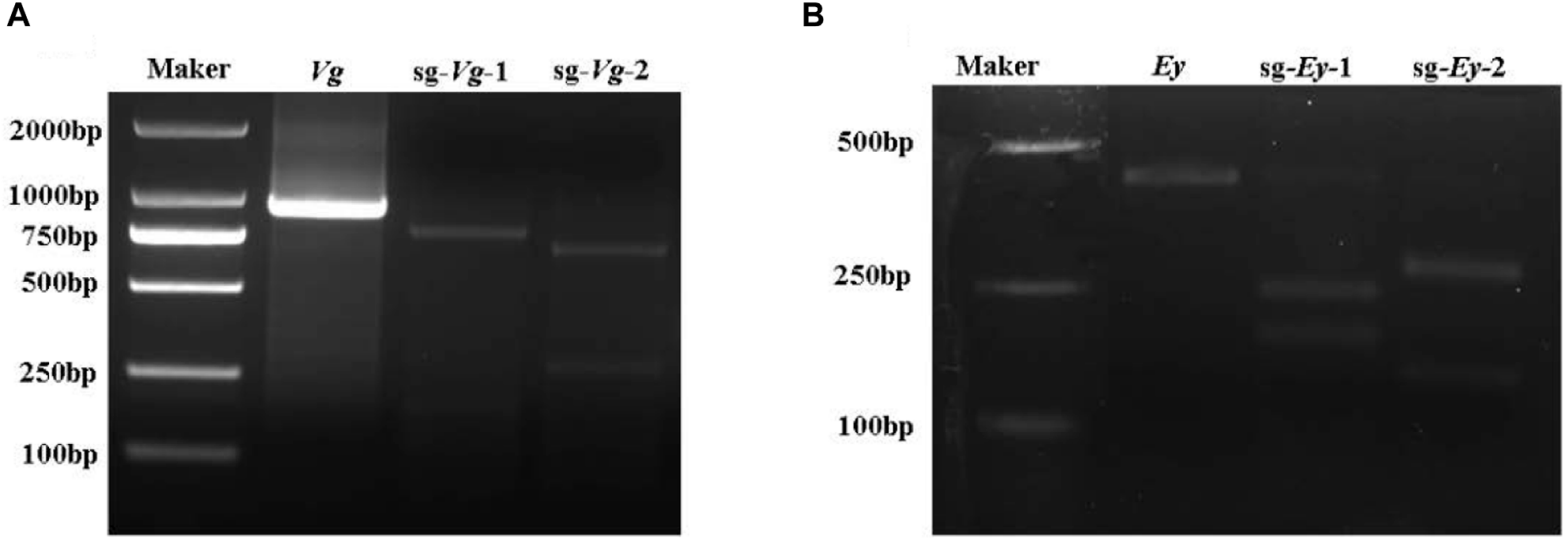
*In vitro* digestion of *Mn-Vg* **(A)** and *Mn-Ey* **(B)** genes.

Two sgRNAs were designed each for *Mn-Vg* and *Mn-Ey* gene editing, named sg-*Vg*-1 and sg-*Vg*-2 and sg-*Ey*-1 and sg-*Ey*-2, respectively. Both sg-*Vg*-1 and sg-*Vg*-2 were designed in the fifth exon of *Mn-Vg*. The two sgRNA loci are shown in Figure 2A, and the sequences are listed in Table 1. A partial 948-bp region including the target regions was amplified to determine the specificity of the two sgRNAs (Figure 4). The results showed that both sg-*Vg*-1 and sg-*Vg*-2 were effective in *in vitro* digestion of the target amplified by the 984-bp sequence. The digestion of sg-*Vg*-1 produced clear bands of approximately 184 bp and 764 bp in the gel. The digestion of sg-*Vg*-2 produced clear bands of approximately 272 bp and 676 bp in the gel.

Both sg-*Ey*-1 and sg-*Ey*-2 were designed in the fifth exon of *Mn-Ey*. The two sgRNA loci are shown in Figure 2B, and the sequences are listed in Table 1. A partial 377-bp region including target regions was amplified to determine the specificity of the two sgRNAs (Figure 4). The digestion of sg-*Ey*-1 produced clear bands of approximately 157 bp and 220 bp in the gel. The digestion of sg-*Ey*-2 produced clear bands of approximately 238 bp and 139 bp in the gel. The digestive efficiency of all sgRNAs exceeded 90% based on the brightness of the bands.

### 3.4 Mutation mediated by CRISPR/Cas9

The survival and mutation rates of key embryo stages mediated by CRISPR/Cas9 were recorded and analyzed (Table 2). For the control group (Sg-*GFP*), among 198 injected one-cell-stage embryos, 105 entered the blastula stage (53.03%). Approximately 16 (8.08%) embryos entered the gastrula stage. Finally, about 5.56% embryos hatched.

**TABLE 2:** Mutation mediated by CRISPR/Cas9.

sgRNA	Number of embryos injected	Blastula				Gastrula		Hatching larva			
		Number of embryos survived	Total embryos survived ratio (%)	Number of mutant sequences (*n* = 30)	Mutant sequence ratio (%)	Number of embryos survived	Total embryos survived ratio (%)	Number of embryos survived	Total embryos survived ratio (%)	Number of mutant individuals	Mutant individual ratio (%)
Sg-*GFP*	198	105	53.03	0	0	16	8.08	11	5.56	0	0
sg-*Vg*-1	207	118	57.00	3	10.00%	18	8.69	10	4.83	3	30.00%
sg-*Vg*-2	188	103	54.78	5	16.67%	11	5.85	6	3.19	0	0
sg-*Ey*-1	128	68	53.13	3	10.00%	8	6.25	3	2.34	2	66.67%
sg-*Ey*-2	150	79	52.67	0	0	9	6.00	4	2.67	0	0

**FIGURE 5:**
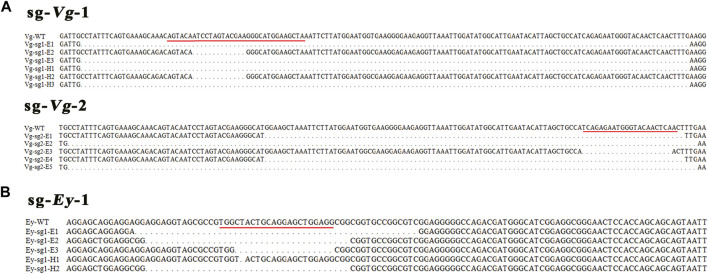
Sequences of the mutations of *Mn-Vg* **(A)** and *Mn-Ey* **(B)** genes in embryos and hatching larvae. The red underline indicates a genomic target site. WT, wild-type; E, embryo; H, hatching larva.

In the *Mn-Vg* group, for sg-*Vg*-1, a total of 207 one-cell-stage embryos were injected, of which about 57.00% (118) of the embryos entered the blastula stage. The gastrula survival ratio was 8.69% (18), and the final hatching ratio was 4.83% (10). Thirty blastula embryos were randomly selected and mixed for DNA extraction. Thirty clones were randomly selected for sequencing, and three mutant sequences were detected (mutant ratio 10%). Among 10 hatching individuals, three mutant sequences were detected (mutant ratio 30%). For sg-*Vg*-2, 54.78% (103) of the 188 injected one-cell-stage embryos entered the blastula stage. The gastrula survival ratio was 5.85% (11), and the final hatching ratio was 3.19% (6). Thirty clones of 30 blastula embryos were randomly selected for sequencing, and five mutant sequences were detected (mutant ratio, 16.67%). No mutant sequences were detected (mutant ratio, 0%) in hatching individuals. All mutant sequencing results are shown in Figure 5A.

In the *Mn-Ey* group, for sg-*Ey*-1, a total of 128 one-cell-stage embryos were processed with microinjection, while 68 (53.13%) of them survived to the blastula stage. The survived ratios of gastrula and hatching larvae were 6.25% (8) and 2.34% (3), respectively. Thirty clones were randomly selected for sequencing based on the mixed DNA of thirty blastula embryos. The results showed that three mutant sequences were recorded (mutant ratio, 10.00%). Two out of three hatching individuals were detected as mutants (66.67%). For sg-*Ey*-2, 52.67% (79) of the 150 injected one-cell-stage embryos entered the blastula stage. The survival ratios of gastrula and hatching larvae were 6.00% (9) and 2.67% (4), respectively. However, no mutant sequence was detected in both blastula-stage and hatching individuals. All mutant sequencing results are shown in Figure 5B.

### 3.5 Phenotype investigation after microinjection

**FIGURE 6:**
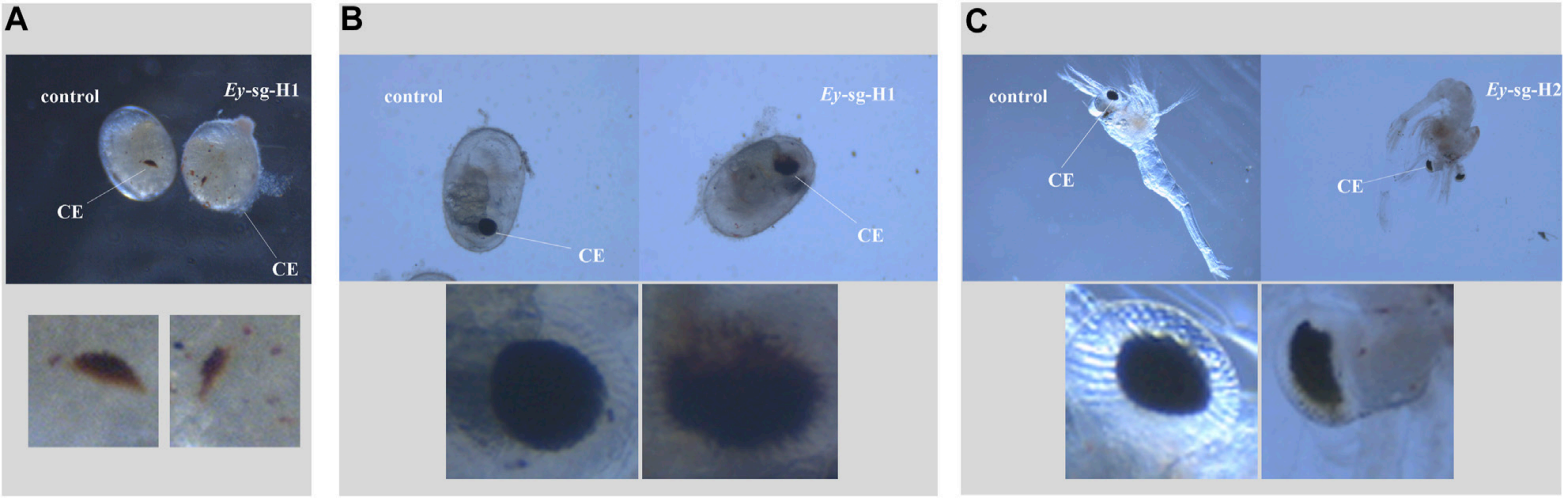
Phenotypic characteristics of injected embryos with sg-*Ey*-1. **(A)** Evident developmental delay of the compound eye was detected in *Ey*-sg1-H1 in the zoea stage. **(B)** Compound eyes of *Ey*-sg1-H2 embryo could not form well-defined spheres and the whole compound eyes appeared to diffuse at the end of the late-zoea stage. **(C)** Half absence of compound eyes was clearly observed in *Ey*-sg1-H2 larvae. CE, compound eye.

The embryo development of both injected and control groups were monitored under the stereomicroscope. In the *Mn-Vg* group, no significant morphological changes were observed. In addition, there was no significant difference in the embryo growth rate between the injected and control groups. In the Mn-*Ey* group, two deformed types were detected in the sg-*Ey*-1 group, but no significant morphological changes were observed in the sg-*Ey*-2 group. Two deformed types detected in the sg-*Ey*-1 group were defined as *Ey*-sg1-H1 and *Ey*-sg1-H2. For *Ey*-sg1-H1 (Figure 6A), the deformity was characterized by delayed development. An evident developmental delay of the compound eye was detected, and the embryo cycle of *Ey*-sg1-H1 was 2 days longer than that of the control group. For *Ey*-sg1-H2, the compound eyes could not form well-defined spheres, and the whole compound eyes appeared to diffuse (Figure 6B). The viability of the *Ey*-sg1-H2 embryo was poor, and it died on the first day after hatching. Compared to the control prawn, a half absence of compound eyes was clearly observed in the *Ey*-sg1-H2 larva (Figure 6C).

## 4 Discussion

CRISPR/Cas9, used for eukaryotic gene editing, has been widely applied to medicine, biology, agriculture, and other fields, since it was first reported in 2013 (Cho et al., 2013; Cong et al., 2013; Mali et al., 2013). It has the characteristics of universality, high specificity, high efficiency, and rapid speed and has a wide application potential in the basic research of aquatic animal genomics and genetic breeding. Recently, the CRISPR-Cas9 system has also been introduced to a few crustaceans, such as model species (*D. magna*, *Parhyale hawaiensis*, and *Exopalaemon carinicauda*) and economic species (*M. rosenbergii*, *N. heteropoda*, and *E. sinensis*) (Nakanishi et al., 2014; Gui et al., 2016; Martin et al., 2016; Li et al., 2022; Molcho et al., 2022). *M. nipponense* is an important economic freshwater prawn; however, the CRISPR/Cas9-based gene-editing system has not been established yet in this species.

In this study, a CRISPR/Cas9-based genomic editing system was established in *M. nipponense* using two different genes: *Vg* and *Ey* genes. In crustaceans, the reproductive regulation mechanism has become a focus of research. *Vg* gene, a precursor of vitellin, plays an important specific role in female gonad maturation (Wilder, 2010). The molecular characteristics and function of *Vg* have been widely studied in crustaceans (Raviv et al., 2006; Okumura et al., 2007; Jia et al., 2013; Chen et al., 2018; Jayasankar et al., 2020). *Mn-Vg* was also functionally clarified by RNA interference and selected as a candidate target gene in controlling the rapid maturation of *M. nipponense* (Bai et al., 2015). *Ey* gene, which is a conserved homolog of the transcription factor paired box protein 6 (Pax6), has been reported to be important for compound eye formation in arthropods (Yang et al., 2009; Kumagai et al., 2017). It was also employed in the crustacean CRISPR/Cas9-based gene editing of *E. carinicauda* (Gao et al., 2020). The CRISPR/Cas9-based genomic editing in this study was successful using both *Vg* and *Ey* genes. However, the two functional genes showed completely different editing effects.

The results of this study are summarized and discussed as follows, which can help improve and promote *M. nipponense* editing technology in the future:

1) An embryonic development schedule was established in *M. nipponense* through the continuous observation of the embryonic development process. The optimal time for microinjection was determined for gene editing based on this schedule. It also supplied the theoretical basis for the stage division of mutagenesis. A more precise time for microinjection during the one-cell stage should be investigated in further studies to improve editing efficiency.
2) Different sgRNAs were designed for the same exon of each gene, and all sgRNAs were tested by *in vitro* digestion. The *in vitro* digestion results showed that there was no large difference in digestion efficiency between two sgRNAs of the same gene; however, the *in vivo* editing results showed that the editing efficiency of different sgRNAs varied significantly. This phenomenon needs to be confirmed by further experiments and research studies, and more sgRNAs are necessary for successful gene editing.
3) The survival ratios and mutant ratios of the two genes were different. Based on the results of the expression profile of an embryo, we found that *Ey* gene was mainly expressed in embryonic development before hatching, while *Vg* gene was mainly expressed after hatching, which was similar to the results of *E*. *carinicauda* (Gao et al., 2020). Combined with the mutant phenotype results, we speculated that: *Ey* gene played an important role in early embryonic development, while the *Vg* gene’s function was not evident in the early development. The observed survival ratio of *Ey* was lower than that of *Vg* and control groups; however, the statistical analysis result showed that there was no significant difference among these groups (*p* > 0.05). Similar results were found in *Ey* gene-editing of *E. carinicauda* (Gao et al., 2020) (lack of statistical analysis). The possible reason was that the deformity of the compound eyes by gene editing increased the mortality rate. Because the crustacean compound eyes were important secretory organs, secreting a variety of important hormones, such as molt-inhibiting hormone, gonad-inhibiting hormone, and crustacean hyperglycemic hormone, which played key roles in development and reproduction (Qiao et al., 2015; Cohen et al., 2021; Zhang et al., 2021). More experiments and data were needed to clarify this phenomenon. The observed survival ratios and mutant ratios in this study were lower than those in *chitinase 4* gene-editing of *E. carinicauda* (Gui et al., 2016) but a bit higher than the ones in *scarlet* gene editing of *N. heteropoda* (Li et al., 2022). The function variation of target genes may also be one of the reasons for differences in survival rates after editing.
4) The sequence mutation rate was higher than that of the phenotype mutation. There was no phenotype mutation observed in *Vg* groups. One hypothesized reason is that the function of *Vg* might not be evident in early embryo development. An alternative hypothesis is that the edited G0 generation was mosaic, which resulted in the retention of part of the target gene function in individual larvae. Mosaic G0 generation without a phenotype mutation has been reported in many aquatic species, such as *Cynoglossus semilaevis*, *Pagrus major*, and *O. latipes* (Cui et al., 2017; Kishimoto et al., 2018; Namgung et al., 2021; Nie et al., 2022). Subsequent research should focus on the hybridization of wild-type *M. nipponense* with mosaic G0 generation to obtain stable G1 with heredity stability.

## Data Availability

The datasets presented in this study can be found in online repositories. The names of the repository/repositories and accession number(s) can be found in the article/Supplementary Material.
